# Primary Squamous Cell Carcinoma of the Descending Colon

**DOI:** 10.7759/cureus.8588

**Published:** 2020-06-12

**Authors:** Nooshin G Jahromi

**Affiliations:** 1 Radiation Oncology, GenesisCare, Brisbane, AUS

**Keywords:** squamous cell carcinoma, descending colon

## Abstract

Primary squamous cell carcinoma (SCC) of the colon is a rare malignancy. The most reported anatomic location is the rectosigmoid colon. In this paper, we report a case of a 74-year-old man with primary SCC of the descending colon treated by surgery and adjuvant chemotherapy. We diagnosed primary SCC of the descending colon because except in the colon, no malignant lesions were found by systemic CT. The role of chemoradiation regimen and duration remains unclear due to the rarity of this entity.

## Introduction

Squamous cell carcinoma (SCC) of the gastrointestinal tract is an extremely rare clinical entity and usually involves the esophagus or anal canal [1]. Few cases of SCC of the colon have been reported in the literature. The most frequently reported location is in the rectosigmoid colon [2]. There are various hypotheses regarding the etiology of colorectal SCC, such as differentiation of a pluripotent stem cell or the squamous metaplasia resulting from external irritation [3,4]. Chronic inflammation or viral infection may also contribute to the development of colorectal SCC [5]. However, the definite etiology of colorectal SCC remains to be determined.

Primary SCC of the colon represents less than 0.5% of all colorectal tumors, with an incidence estimated at 0.1% in the literature [3]. Clinical manifestations, biological behavior, treatment, and prognosis remain largely unknown. In this paper, we report a case of primary SCC of the descending colon.

## Case presentation

A 74-year-old man presented with a three-month history of left-sided lower abdominal pain and associated weight loss. Physical examination revealed bilateral inguinal hernias, which were all reducible, and a non-tender palpable mass in the left side of the abdomen. The patient had no history of cancer or a family history of colonic malignancy.

**Figure 1:**
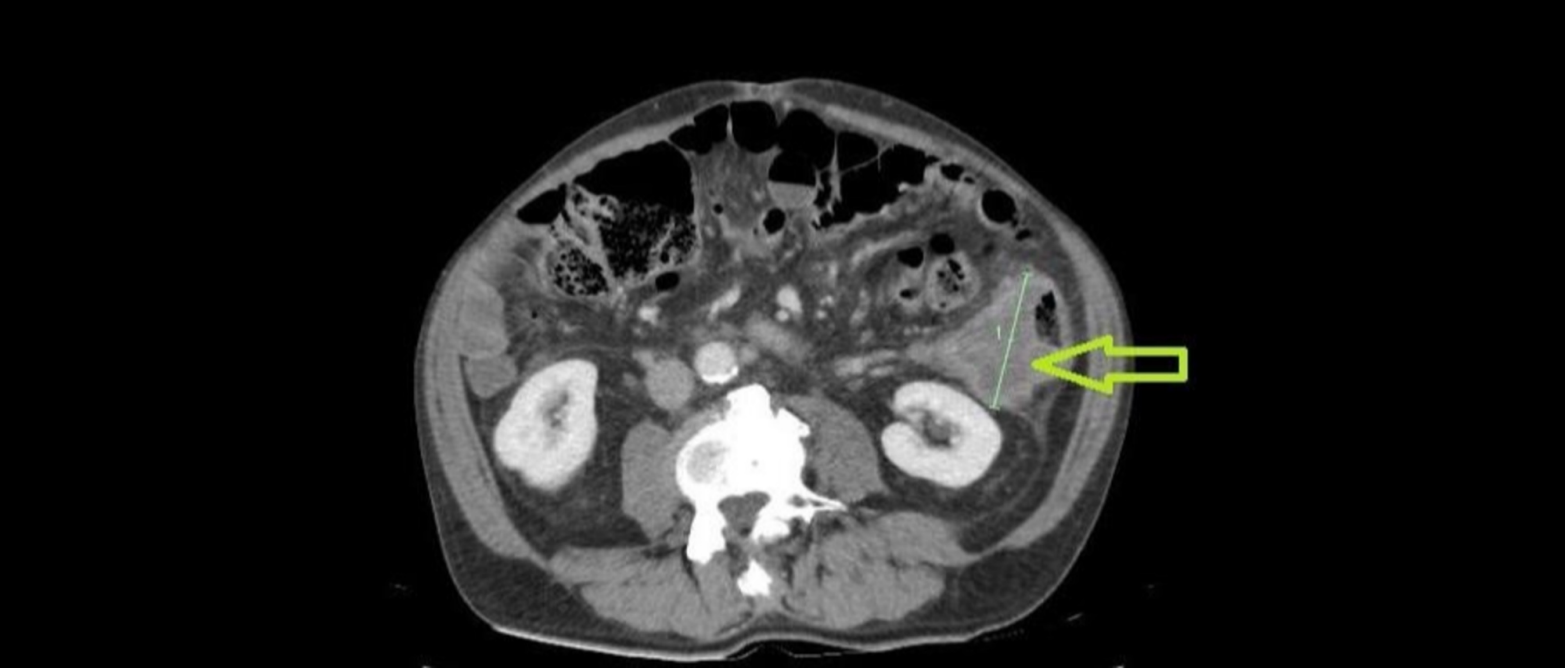
Abdominal CT scan post-contrast showing a large exophytic solid mass arising from the mid-descending colon, measuring approximately 7 cm x 6.5 cm x 6 cm.

**Figure 2:**
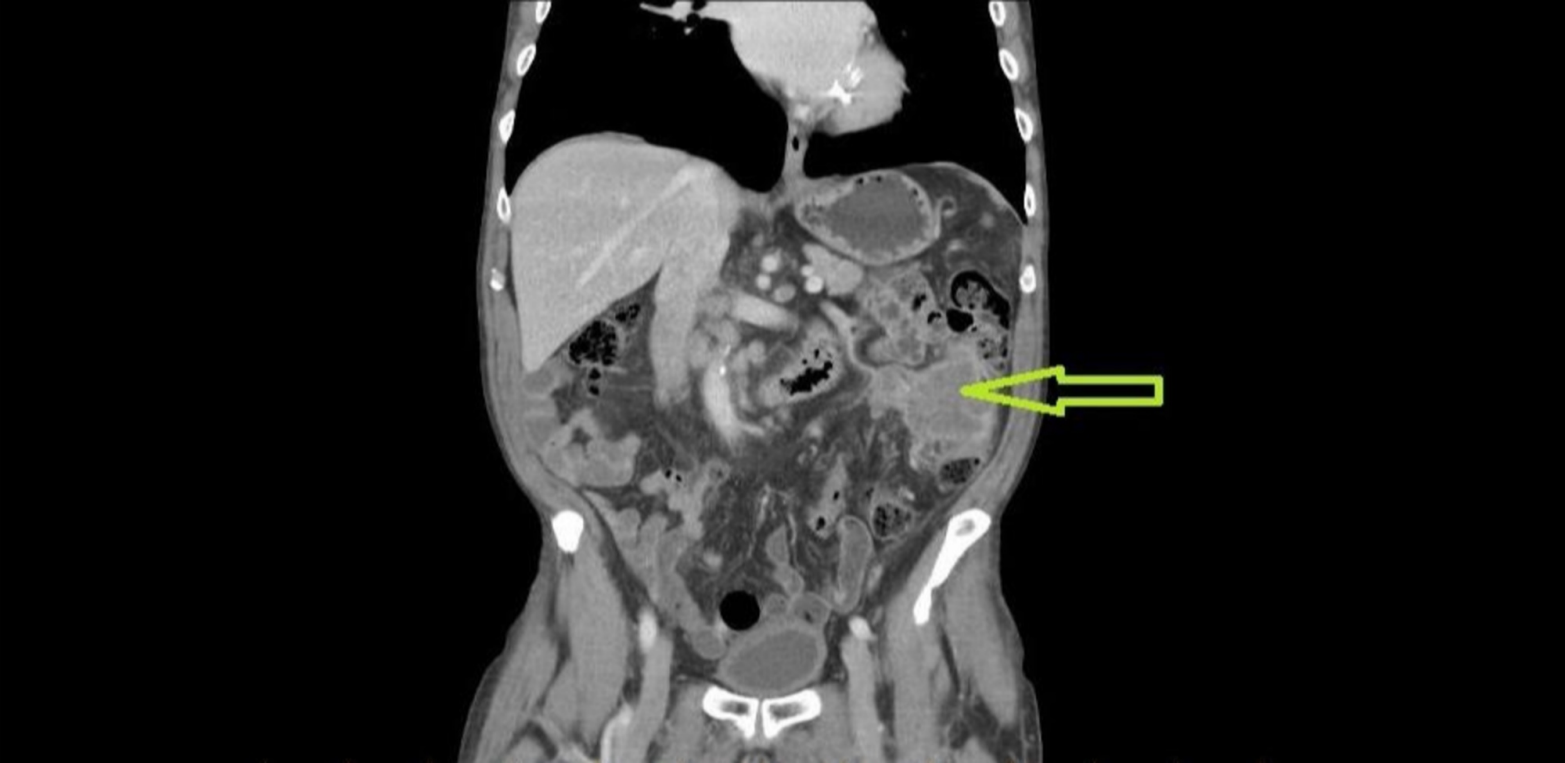
Coronal CT scan post-contrast revealing a colonic mass with medial extension into the mesentery.

CT scan showed a large exophytic colonic mass arising from the mid-descending colon, measuring 7 cm x 6.5 cm x 6 cm with medial extension into the mesentery with multiple enlarged lymph nodes in the retroperitoneum and retrocrural region (Figures 1, 2). Colonoscopy demonstrated a circumferential fungating mass in the descending colon. His body scan did not show any primary source of SCC. His PET scan revealed retroperitoneal, periportal, retrocrural, and posterior mediastinal nodal metastases.

The patient underwent laparoscopic left hemicolectomy, which revealed an advanced descending colon tumor extending into Gerota’s fascia. Histological examination was consistent with a poorly differentiated SCC with evidence of extramural venous and lymphovascular invasion. There were 3/12 positive lymph nodes that were pathologically staged as pT3 and pN1b (stage IIIB). The patient was referred to a medical oncologist for three to four cycles of palliative systemic chemotherapy with FOLFOX (folinic acid, fluorouracil, and oxaliplatin).

## Discussion

It is very rare that SCC arises from the colorectal epithelium [6]. Schmidtman published the first report of a pure SCC of the colon in 1919 [6]. Since then, less than 150 cases have appeared in the literature [7]. Most pure SCC cases of the colon have been reported in the rectosigmoid colon, where as our case it was in the descending colon. SCC of the colon occurs most commonly around the fifth decade of life, with a slight male predominance [8]. Clinically, it can present with similar signs and symptoms as colorectal adenocarcinoma with a diagnostic delay between 6 weeks and 12 months [9].

Miyamoto et al. suggested that certain criteria are required before a diagnosis of primary SCC of the colon is made [6]. First, metastasis from other sites to the bowel must be excluded. Second, a squamous-lined fistulous tract must not involve the affected bowel, as this can be a source of SCCs. Third, SCCs of the anus with proximal extension must be ruled out. Fourth, histological analysis must confirm the SCC.

Assessing the prognosis of patients with colorectal SCC is challenging due to its rarity. Certain factors would be associated with a poor prognosis such as the ulcerated nature of the lesion, the left localization of the tumor, lymph nodes metastasis, stage IV of TNM, and the degree of differentiation of the tumor (poorly differentiated and undifferentiated) [7].

The colorectal SCCs appear to be more frequently locally invasive and more likely to involve regional lymph nodes than the adenocarcinomas, probably due to delayed diagnosis [6]. In this case, the tumor was pT3 with lymph node involvement. Left hemicolectomy with a negative resection margin was performed.

The optimal treatment for SCC has not been determined due to its low incidence. Generally, the treatment is based on that for adenocarcinomas [10]. The efficacy of adjuvant chemotherapy or radiation and duration has not been established given the rarity of the tumor. In a study by Miyamoto et al., surgical resection and adjuvant chemotherapy were considered better approaches to the treatment of colorectal SCC [6]. Zhao et al. reported that SCC patients with stage III-IV disease have a poorer prognosis compared with those with colonic adenocarcinoma [10]. As a result, a more aggressive chemotherapeutic approach may be a feasible choice for patients with a good performance status. In their study, gemcitabine was recommended as a treatment option for colon SCC in the neoadjuvant and/or adjuvant chemotherapy setting.

Copur et al. concluded that cisplatin, etoposide, and 5-fluorouracil as a combination chemotherapy was useful for colorectal SCC [8]. Juturi et al. suggested 5-FU, cisplatin, and leucovorin as a combination therapy in metastatic SCC of the colon [11].

In this case, we thought that it would be reasonable to offer three to four cycles of FOLFOX chemotherapy as this regimen is active in esophageal SCC. However, the repeat CT scan showed the progression of nodal disease consistent with progressive colon carcinoma and new adrenal metastasis.

The role of radiotherapy remains unknown, but a radiotherapy treatment is recommended in cases with post-operative positive margins.

## Conclusions

We presented a case of primary SCC of the descending colon in a 74-year-old man. SCC of the colon is exceptionally rare; therefore, clinical manifestations, treatment, and prognosis remain poorly defined. However, we believe that surgical resection and adjuvant chemotherapy may have a role in most cases.
